# Establishment of an early liver fibrosis model by the hydrodynamics-based transfer of TGF-β1 gene

**DOI:** 10.1186/1476-5926-6-9

**Published:** 2007-10-19

**Authors:** Kun-Lin Yang, Kuo-Chen Hung, Wen-Teng Chang, Eric IC Li

**Affiliations:** 1 Institute of Basic Medical Sciences College of Medicine, National Cheng Kung University, Tainan 701, Taiwan; 2 Department of General Surgery, E-DA Hospital, I-Shou University, Kaohsiung 824, Taiwan; 3 Department of Biological Science and Technology, Chung Hwa University of Medical Technology, Tainan 717, Taiwan; 4 Department of Pharmacology, College of Medicine, National Cheng Kung University, Tainan 701, Taiwan

## Abstract

**Background:**

Liver fibrosis represents a significant and severe health care problem and there are no efficient drugs for therapy so far. Preventing the progression of fibrogenesis and revival endogenous repair activities is an important strategy for both current and future therapies. Many studies of liver fibrosis consist of animal testing with various hepatotoxins. Although this method is often used, the model at which cirrhosis or extensive fibrosis becomes irreversible has not been well defined and is not representative of early-stage fibrogenesis. We here report the establishment of a transient and reversible liver fibrosis animal model which may better represent an early and natural fibrotic event. We used a high-speed intravenous injection of naked plasmid DNA of transforming growth factor-β1 (TGF-β1) gene which is under the control of a metallothionein-regulated gene in a pPK9A expression vector into the tail vein (the hydrodynamics-based transfer) and fed the mouse with zinc sulfate (ZnSO_4_)-containing water simultaneously.

**Results:**

Using our hydrodynamics-based gene transfer model we found that upon induction by ZnSO_4_, the serum TGF-β1 level in Balb/c mice and Sp1 transcription factor binding activity peaked at 48 h and declined thereafter to a normal level on the 5^th ^day. In addition, mRNA and protein levels of TGF-β1 in the liver were also upregulated at 48 h. Furthermore, induction of TGF-β1 increased the α-smooth muscle actin (α-SMA), p-Smad2/3, hydroxyproline and collagen 1A2 (Col 1A2) levels in the liver, suggesting a significant liver fibrosis.

**Conclusion:**

Our results show that TGF-β1 in pPK9a-transferred mice liver with ZnSO_4 _feeding can achieve a high expression level with significant fibrosis. However, since TGF-β1 induction is transient in our model, the fibrotic level does not reach a large scale (panlobular fibrosis) as seen in the CCl_4_-treated liver. Our model hence represents a dynamic and reversible liver fibrosis and could be a useful tool for studying early molecular mechanism of fibrogenesis or screening of antifibrotic drugs for clinical use.

## Background

The development of liver fibrosis, particularly in the cirrhosis stage, is associated with high morbidity and mortality rates [1] and at present the only curative treatment for end stage liver cirrhosis is organ transplantation. The point at which cirrhosis or extensive fibrosis becomes irreversible has not been well defined [2], however, since liver fibrosis is a continuous process in both gene expression and histopathological alterations [3]. Generally accepted animal testing of liver fibrosis includes treatments with hepatotoxins such as carbon tetrachloride (CCl_4_). However, after the cessation of the long-term treatment of CCl_4 _for more than 4 weeks, pathological changes in the liver, such as inflammation, are reversed with the exception of fibrosis [3]. Many experimental *long-term *treatment models of liver fibrosis leading to cirrhosis have been useful for testing drug effectiveness but further studies are required to account for effects of disease treatment when gene expressions, especially TGF-β1 gene, has not yet been irreversibly altered [4].

TGF-β1, a 25-kD multifunctional cytokine, has been demonstrated in a number of animal models to play a major role in the pathogenesis and progression of fibrotic disease [5]. Over expression of TGF-β1 presents not only an early gene change in liver fibrosis but also a direct connection between oxidative stress and collagen upregulation in the fibrosis event [6-8]. Hepatic fibrosis results from a net increased synthesis and decreased degradation of extracellular matrix (ECM) proteins, whose most prevalent protein is Type 1 collagen (Col 1A2). TGF-β1 regulates ECM accumulation in the liver via the generation of reactive oxygen species (ROS) which stimulates calcium (Ca^2+^) influx and induces the activation and contraction of hepatic stellate cell (HSC) [8]. The activated HSC in turn secretes TGF-β1, further augmenting the autocrine regulating cycle.

Another involved pathway is the activation of Smad cascade. The Col 1A2 gene expression is induced via the phosphorylation of Smad2 and Smad3, a Smad containing complex is subsequently translocated into cell nucleus [9]. Studies have shown that synergistic cooperation between Sp1 and Smad3/Smad4 is required for the TGF-β1 response to the collagen gene expression and Sp1 is found to play a critical role in the constitutive expression of Col 1A2 [10]. Cross-talk perhaps exists between these two pathways [8].

The goal of our current investigation is to establish a liver fibrosis model in which the spontaneous reversal of fibrosis is made possible at an early phase. We used a metallothionein-regulated TGF-β1 expression vector (pPK9a) in which the fused TGF-β1 gene is under the control of an inducible metallothionein promoter [11]. This inducible system has been reported to achieve a high-level of transgene expression in the liver when the system is accompanied with the concurrent presence of heavy metal [12]. Hydrodynamics-based gene delivery has attracted a lot of attention in recent years [13]. This procedure involves a large-volume and high-speed intravenous injection of naked plasmid DNA into the animal tail vein; the procedure represents an efficient, simple and convenient transfection method for laboratory animals. The method especially allows the achievement of a high expression level of exogenous gene in liver [12-15]. Combining a hydrodynamics-based gene delivery system and the metallothionein-regulated pPK9a vector, we have established a dynamic mouse liver fibrosis model. In this model the level of TGF-β1 gene can be overexpressed with the presence of zinc sulfate (ZnSO_4_) in the drinking water. In induced state Col 1A2 and α-SMA, the two indicators of fibrosis and HSC activation, are also upregulated. This model could be useful for studying the initial stages of liver fibrosis.

## Results and Discussion

### Expression of TGF-β1 gene in hydrodynamics-based gene transferred mice

**Figure 1:**
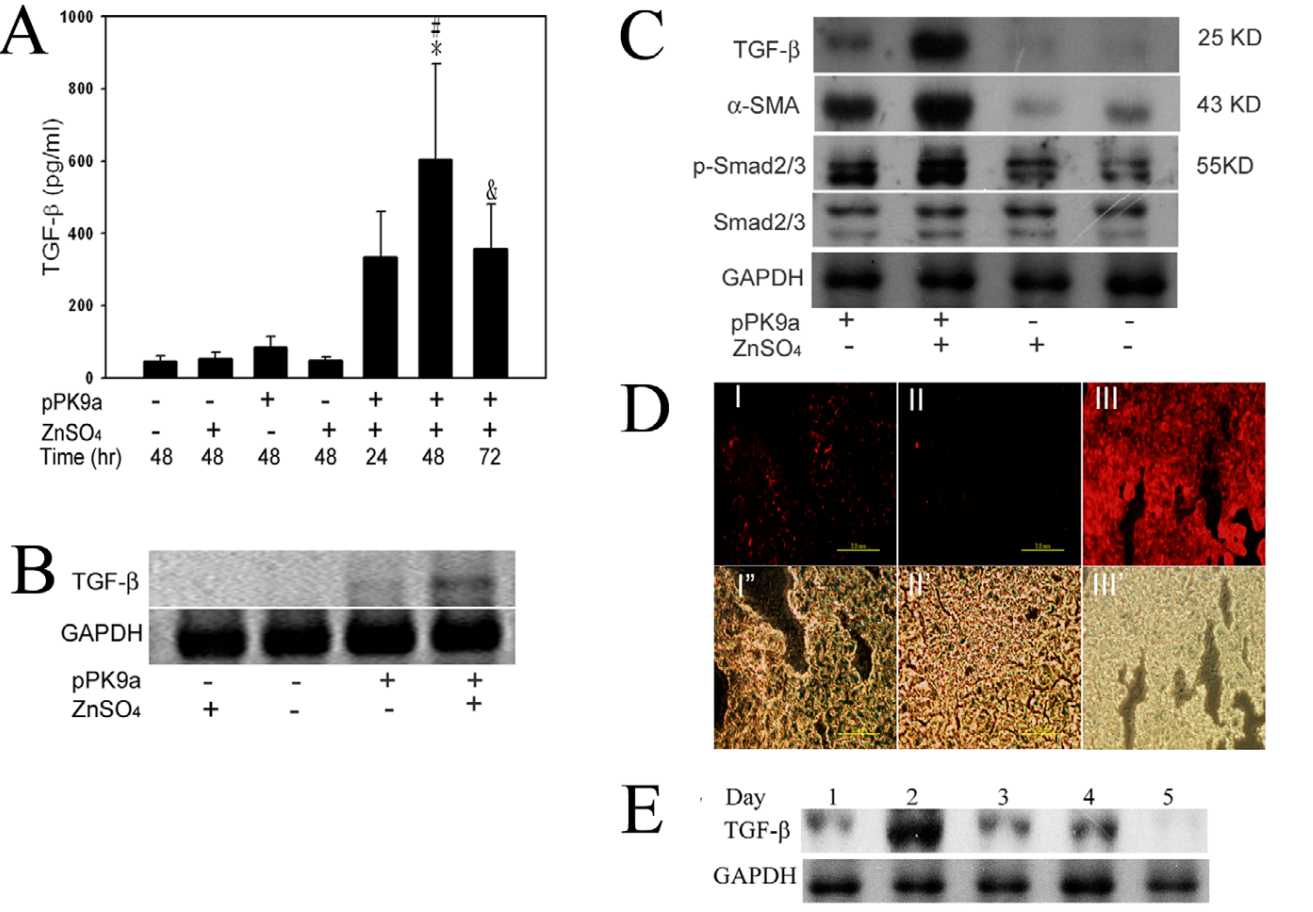
**Conditional regulation of TGF-β1 expression in mice**. (A) Serum TGF-β1 levels in hydrodynamics-based gene transferred mice. Values are represented as mean ± SD. *, p < 0.01; compared with pPK9a alone (48 h); #, p < 0.05; compared with pPK9a + ZnSO_4 _(24 h); and, p < 0.05; compared with pPK9a + ZnSO_4 _(48 h); unpaired *t*-tests. (B) RT-PCR analysis for TGF-β1 mRNA expression at 48th h in the liver of gene transferred mice after injection with pPK9a. (C) Protein expression of TGF-β1 and α-SMA at 48th h in gene transferred mice after injection with pPK9a. (D) Photomicrographs of immunohistochemical analysis (upper panel) and bright field (lower panel) for TGF-β1 expression at 48 h in liver sections: I. Ringer's solution + pPK9a and ZnSO_4_free. II. Ringer's solution only. III. Ringer's solution + pPK9a + ZnSO_4_. Bar = 0.2 mm. (E) TGF-β1 protein expression between 1 to 5 days.

The level of TGF-β1 was assessed by using four independent methods: analysis of TGF-β1 in plasma (Fig. 1A), mRNA (Fig. 1B) and protein (Fig. 1C) in the liver and immunohistochemical staining in liver sections (Fig. 1D). The results indicate that serum levels of TGF-β1 in pPK9a-transferred mice fed with ZnSO_4 _peaked at 48 h and were higher than the four control groups (Fig. 1A). In the absence of ZnSO_4 _and pPK9a the serum TGF-β1 level is much lower than in their presence (Fig. 1A). The serum TGF-β1 values fell between 600 and 900 pg/ml and were 5 to 15 times higher than the controls (Fig. 1A); the peak was followed immediately by a decline at the 72^nd ^h even when ZnSO_4 _was not withdrawn. The level of TGF-β1 declined to its normal level on the 5th day and was no longer inducible on the 7th day (Fig 1E). The results are basically similar to that described by Herweijer et al. who showed time course of gene expression after plasmid DNA gene transfer to the liver: expression of the transferred gene was very high on day1 after portal vein injection of plasmid but diminished quickly by day 2 and declined to low level after day 4 [16]. Their induction was apparently transient as ours. Moreover, Clouthier et al. demonstrated that upon TGF-β1 gene transfer the mice showed similar pathological morphology in both liver and kidney [17]. As observed by them we also find high expression of TGF-β1 in the kidney (data not shown), although in this study we just report our findings on fibrotic events in the liver.

Measurements of the other three analyses were taken at the 48th h and similar results were seen in mRNA and protein expressions in liver tissue (Fig. 1B, C and 1D). Primers specific for porcine TGF-β1 in pPK9a were used for distinguishing transcripts from endogenous mouse TGF-β1 by RT-PCR assay (Fig. 1B). TGF-β1 mRNA was detected in mice with pPK9a-transfer (Fig. 1B lane 1 and 2) but not in those without (Fig. 1B lane 3 and 4). However, in western blot a very trace amount of protein was detected in mice without pPK9a-transfer (Fig. 1C, lane 3 and 4). This could be due to fact that the antibody used in the experiment can recognize TGF-β1 from mice, human and porcine. This trace amount of protein was barely detected in immunohistochemical staining of the liver sections (Fig. 1D II).

The high level of TGF-β1 was accompanied by a strong activation of HSC, as indicated by high expressions of α-SMA and p-Smad 2/3 (Fig. 1C, lane 2). Prominent bands of α-SMA and p-Smad 2/3 were also detected in pPK9a-transferred mice that ingested water in the absence of ZnSO_4 _(Fig. 1A, lane 3; 1B, lane 3; 1C, lane 1; and 1D I). It was reported that a very low level of cadmium is consumed from the diet and this metal ion can induce endogenous metallothionein to express to some extent [18]. We suspect that the cadmium might cause the endogenous metallothionein induction as shown in Fig. 1C (lane 1). However, the effect of ZnSO_4 _on pPK9a became more drastic as shown in Fig. 1D where TGF-β1 was barely detected in D I (without ZnSO_4_) as compared to the brilliant staining in D III (with ZnSO_4_). The levels of TGF-β1 expression in liver pPK9a-transferred mice treated with ZnSO_4 _were upregulated at day 2 and declined at day 3–5 (Fig. 1E). Taken together, the results indicated that TGF-β1 can be markedly induced in pPK9a-tranferred mice treated with ZnSO_4_.

### Histological and immunohistochemical analyses in hydrodynamics-based pPK9a transferred mice

**Figure 2:**
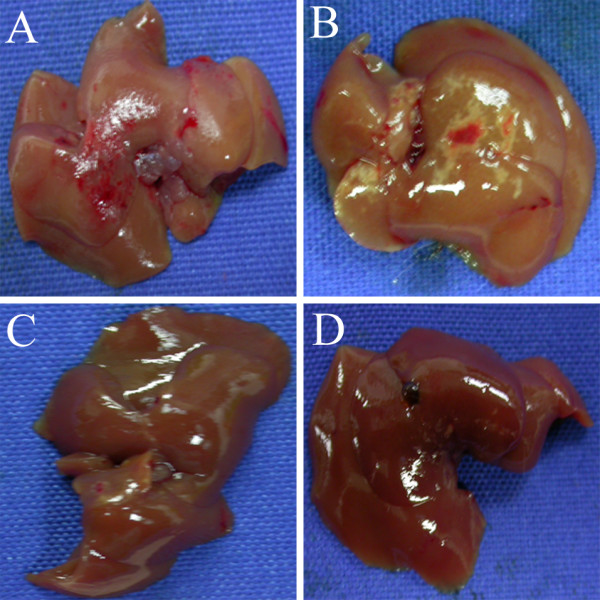
**Observation of the liver**. Livers were obtained from mice treated with Ringer's solution + pPK9a + ZnSO_4 _for 24 h (A), Ringer's solution + pPK9a + ZnSO_4 _for 48 h (B), Ringer's solution + pPK9a for 48 h (C), and vehicle (injection free) for 48 h (D).

**Figure 3:**
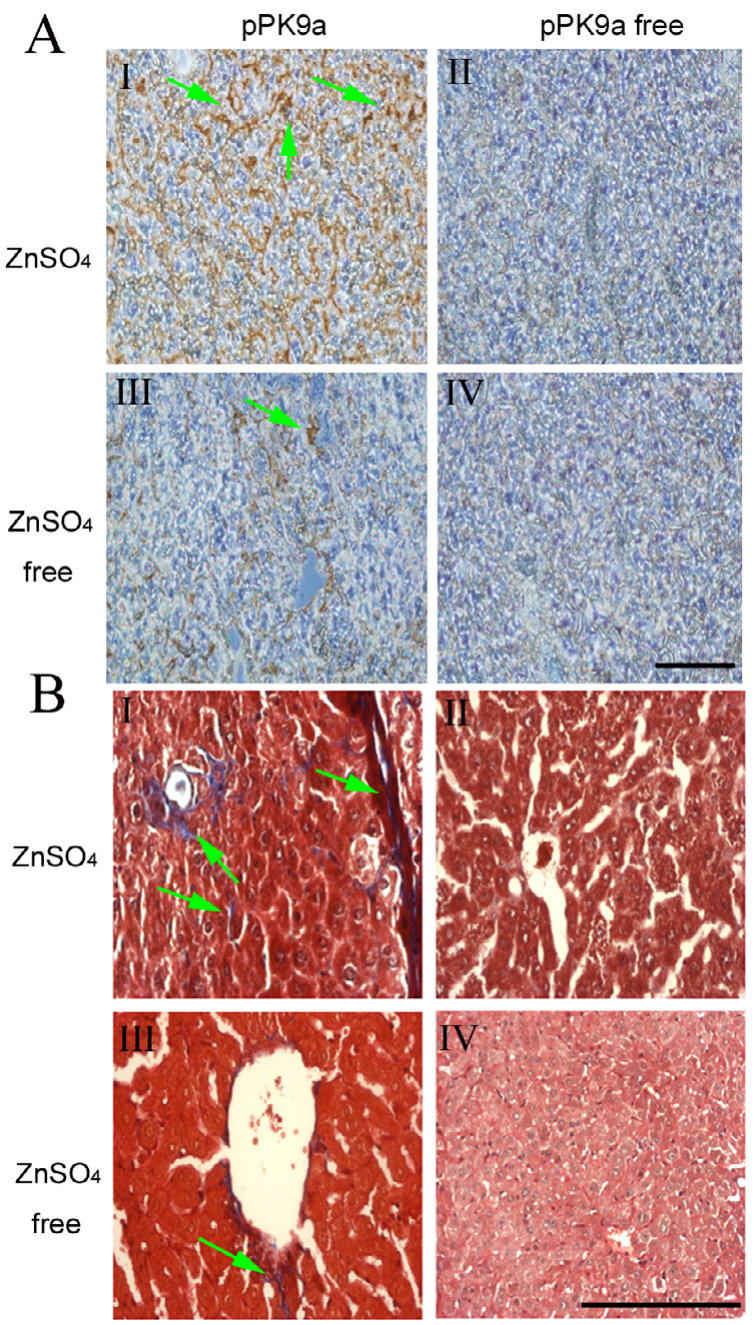
**Upregulation of α-SMA and ECM by gene transfer and ZnSO4 treatment in liver**. (A) Detection of α-SMA by immunohistochemistry. Forty eight h after hydrodynamics-based injection of pPK9a, the mice were sacrificed and the liver sections were subjected to immunostaining. Dark brown granules represent α-SMA signals stained by α-SMA-specific antibody and indicated by arrows. (B) Detection of ECM and collagen by Masson's trichrome staining. The cytoplasm was stained red and collagen fibers in ECM were blue-green. The collagen signals were indicated by arrows. Representative liver sections of α-SMA and collagen from experimental I-IV groups: (I) Ringer's solution + pPK9a + ZnSO_4 _48 h. (II) Ringer's solution + ZnSO_4_. (III) Ringer's solution + pPK9a. (IV) Mice without hydrodynamics-based injection. Bar = 0.2 mm.

Liver sections were sampled from mice with pPK9a transfer at the 24th and 48th h with water containing ZnSO_4 _(24, 48 h; Fig. 2A and 2B). The liver samples looked paler and stiffer than that of the control (Fig. 2C and 2D). Using Masson's trichrome staining, we noticed the liver sections from mice with pPK9a transfer and ZnSO_4 _induction exhibited a marked perisinusoidal deposition of ECM found mostly in the direct vicinity of large vessels (Fig. 3B, I) and a distinct activation of HSC was also observed as shown by α-SMA expression (Fig. 3A, I). The distribution and intensity of collagen and α-SMA were different from that of the control livers where the two proteins were barely detected (Fig. 3). The sinusoids with an enlarged diameter were observed in Fig. 3B I–III, which was not seen in the control (Fig. 3B, IV). However, a bright red patch is noticeable in TGF-β1-overexpressed liver (Fig. 2B), which appears to be hemorrhagic (Fig. 2B). The "hemorrhage" was observed in most of our livers overexpressing TGF-β1 gene. A similar situation of liver hemorrhage was observed by Clouthier et al. who reported that overexpression of TGF-β1 gene in mice caused not only severe liver fibrosis but also in the extreme case hemorrhage and thrombosis [17]. They attributed the extreme situation to the results of overexpression of TGF-β1 and not the triggering cause of TGF-β1 overexpression. We agree with their proposal because we observed a quick increased expression of TGF-β1 followed by a quick decline. Should the TGF-β1 expression was caused by liver damage, we would not have found a prompt decline of TGF-β1 (Fig. 1A and 1E). More research is definitely needed to clarify this controversy.

### Serum biochemical analysis

**Figure 4:**
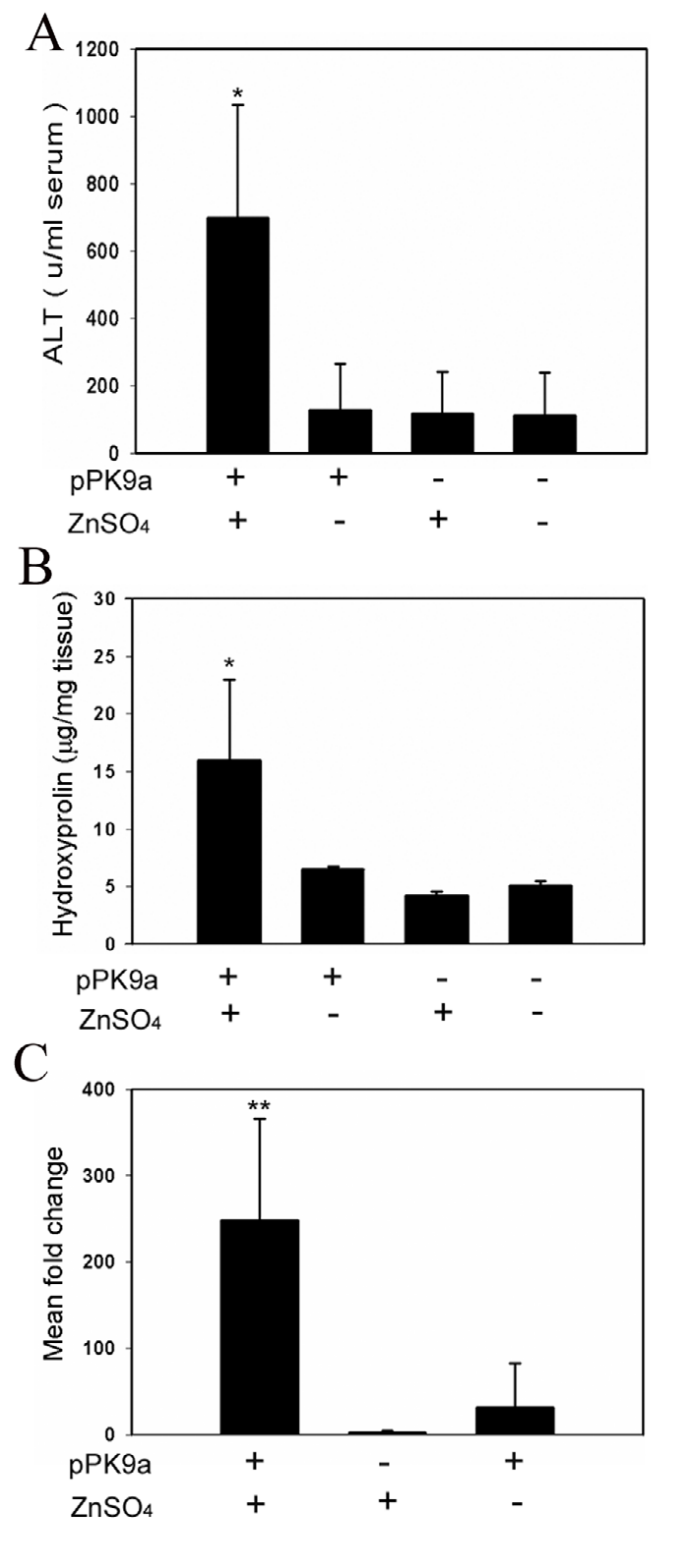
**Observation of liver fibrosis in transgenic mice**. (A) Serum ALT levels. (B) Hydroxyproline content in liver. (C) Col 1A2 mRNA levels measured by real-time quantitative PCR. All samples were collected 48 h after gene transfer and induction with ZnSO_4_. Values are represented as mean ± SD. *, p < 0.05; **, p < 0.001; compared with other groups; unpaired *t*-tests.

Forty eight hours after a hydrodynamics-based injection of pPK9a, TGF-β1 was induced by ZnSO_4 _and triggered a hepatic injury (Fig. 2 and 3B), resulting in increased alanine transaminase (ALT) levels in the serum of approximately 6 times higher than that of the control groups (Fig. 4A).

### Collagen expression in hydrodynamics-based fibrosis in mice

The degree of fibrosis was assessed by using three independent methods: the collagen quantitation by measuring hydroxyproline content (Fig. 4B), Col 1A2 mRNA level in liver samples (Fig. 4C) and the histopathological analysis under light microscope (Fig. 3B). The results provide factual evidence that the pPK9a-transferred mice upon induction by ZnSO_4 _could strongly elicit the expressions of hydroxyproline and Col 1A2 over 3 folds and 200 folds, respectively, as compared to the normal control groups (Fig. 4B, C).

HSC activation plays a key role in liver fibrosis at the early phase and activated HSC is accompanied with high expressions of p-Smad2/3 and α-SMA proteins [9,10,19,20]. Our results mirror this fact as shown in Fig. 1C where both proteins are markedly expressed as compared with the controls. Cirrhosis represents a later stage of progressive scarring in chronic liver disease; it begins with subendothelial or pericentral fibrosis (hepatic fibrosis) and progresses to panlobular fibrosis with nodule formation (cirrhosis) [2]. Our study demonstrates that liver TGF-β1 of pPK9a-transferred mice with ZnSO_4 _feeding can achieve a substantial increased expression level with fibrosis. However, since our TGF-β1 expression is transient, the fibrotic level does not reach a large scale (panlobular fibrosis) as seen in the long term CCl_4_-treated liver [Additional file 1]. Although this CCl_4_-induced cirrhosis model is commonly used, its effect is systemic and no attempts are made to clarify the influence of CCl_4 _toxicity [3]. In this regard, our model is apparently different from the CCl_4 _model with respect to TGF-β1; the life of TGF-β1 is transient, dynamic and overexpressed. We also noticed that the transient overexpression of TGF-β1 in the liver leads to an increased deposition of ECM around the vessels as well as along the sinusoids (Fig. 3B). This finding is consistent with the observation described by Ueberham et al. on transgenic animal models [21].

### Gel electrophoretic mobility shift assays (EMSA) for Sp1 protein

**Figure 5:**
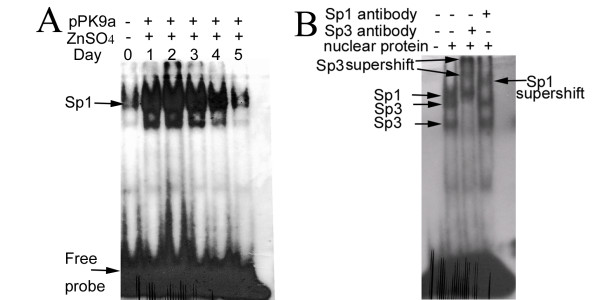
**Enhancement of Sp1 binding activity by gene transfer accompanying with ZnSO_4 _treatment**. (A) Measurement of Sp1 binding activity performed by EMSA. Nuclear proteins were extracted from the livers of pPK9a-transferred mice administrating ZnSO_4_-contained water for 1–5 days. Two μg of nuclear extract was subjected to ^32^P-labelled probe and the Sp1-DNA complex was analyzed on a 4% native polyacrylamide gel. (B) Confirmation of Sp1 and Sp3 binding by supershift assay. Sp1 and Sp3 antibodies were added to the reaction mixtures for supershift assays. The shifted and supershifted bands were indicated by arrows.

To probe into the down steam effectors of TGF-β1 we refer to the EMSA assay to see if Sp1 molecule specifically is involved in the signaling pathway. TGF-β1 being a strong activator of ECM accumulation stimulates the Col 1A2 gene expression by inducing the binding of a Sp1- and p-Smad2/3-Smad4-containing complex to Col 1A2 upstream promoter element (-330 bp to -286 bp and -271 bp to -255 bp; TGF-β1 responsive element; TbRE) which contains a CAGA box. Since Sp1 is a critical mediator of Col 1A2 expression, we deem it prudent to examine if Sp1 was induced in pPK9a-transferred mice treated with ZnSO_4 _and hence performed the supershift assay to confirm the Sp1 and Sp3 binding to TbRE. Fig. 5 shows that the binding activity of Sp1 in liver increased at day 2 and decreased at day 3–5. The pattern of Sp1 binding strongly correlates with the expression levels of TGF-β1 (Fig. 1E). Since it has been reported that Sp1 is required for the *early *response of Col 1A2 to TGF-β1 and maintenance of the constitutive expression of Col 1A2 [20], our results provide direct evidence confirming that our pattern of fibrosis model is early, dynamic and reversible. Moreover, Fig. 3A and 4C show that temporal activation of TGF-β1 and Sp1 is correlated with α-SMA and Col 1A2 expressions, a finding consistent with previous reports [19,20].

## Conclusion

The important role played by TGF-β1 in liver fibrosis has been well documented [5,7,8] and has been shown in transgenic mice model using pronuclear injection by standard technique [21]. What we have shown here is a rapid fibrosis model with transient and reversible over expression of TGF-β1 and Sp1 transcription factor. The expression is hence an early event. We infer that the fibrosis might also be transient and reversible. However, the expression of transferred TGF-β1 appears to be systemic, not restricted to the liver as has been reported by our observation and by others. But since hydrodynamic gene transfer coupled with metallothionein promoter and ZnSO_4 _induction has been reported to have higher expression level of the exogenously delivered gene in the liver [12-15], our model is more unique to the liver and may have its usefulness for the clinical study of the prevention of early stage of liver fibrosis.

## Methods

### Animals

Mice of Balb/c strain were used. All procedures of animal handling were approved by the Institutional Animal Care and Use Committee of National Cheng Kung University. Eight-week-old mice were used in all experiments and were divided into five groups (*n *= 21), fed *ad libitum *standard laboratory feed and water with or without 25 mM ZnSO_4 _plus 5% sucrose in the animal facility [22].

### cDNA construction

TGF-β1 cDNA was constructed in pPK9a vector and was under the regulation of metallothionein promoter. Cys^223 ^and Cys^225 ^in the TGF-β1 pro-peptide were also converted to serine, a mutation that results in dissociation of the pro-peptide and secretion of bioactive TGF-β1 [11]. It has been found that this mutation does not alter TGF-β1 production but does yield a high proportion of mature 25 kDa dimer which is bioactive without acid activation [11,22]. pPK9a was a gift from Professor Paturu Kondaiah of the Indian Institute of Science, India.

### Amplification and purification of plasmid DNA

The plasmid used for administration was purified with a purification kit (Qiagen, Hilden, Germany) according to the manufacturer's instructions.

### Hydrodynamics-based transfection

Ten μg of plasmid (pPK9a) were dissolved in 3.0 ml Ringer's solution (NaCl 0.154 M, KCl 5.63 mM, and CaCl_2 _2.25 mM) and injected into the mouse tail vein in a short duration of 5–7 s according to the hydrodynamics-based transfection protocol as described [14]. ZnSO_4 _(25 mM) was dissolved in the drinking water to activate the metallothionein promoter and stimulate TGF-β1 expression [22,23].

### Serum TGF-β1 levels

Blood was gathered by puncturing into retro-orbital veins with a 27 gauge needle and reserved in a tube for 30 min at 4°C. Serum was separated by centrifugation at 2,640 *g *for 3 min at 4°C. TGF-β1 levels were determined by enzyme-linked immunosorbent assays (ELISA) method (DuoSet ELISA, R&D Systems, Minneapolis, MN).

### Induction of TGF-β1 expression in liver

RNA was isolated from liver tissue by using Trizol Reagent (GIBCO Life Technologies, Rockville, MD). TGF-β1 mRNA expression was detected by means of RT-PCR with specific primers that distinguish porcine TGF-β1 transcript from that of the mouse endogenous TGF-β1. PCR primers specific for porcine TGF-β1 were: 5'-GAAAGCGGCAACCAAATC-3' and 5'-TGACATCAAAGGACAGCCAC-3'. Additional primers used for RT-PCR of glyceraldehydes-3-phosphate dehydrogenase (GAPDH) gene, were 5'-CCCTTCATTGACCTCAACTAC-3' and 5'-CCACCTTCTTGATGTCATCAT-3'. All RT-PCR reactions were done for 35 cycles [24].

### Western blot analysis

For studying protein expressions of TGF-β1 and α-SMA, liver tissue was homogenized in a RIPA buffer (50 mM Tris-HCl, pH 8; 150 mM NaCl; 1%

Nonidet P-40; 0.1% SDS; 1% Triton X-100 plus protease inhibitors Sigma, St. Louis MO) and centrifuged as described [25]; supernatant was taken as a whole-cell lysate. TGF-β1 and α-SMA were electrophoresed under non-reducing condition on a 12% SDS-polyacrylamide gel, transferred by electroblotting to a PVDF membrane, and visualized by immunostaining. Anti-TGF-β1, anti-phospho-Smad 2/3 (Ser433/435-phosphorylated Smad2/3; p-Smad2/3), anti-Smad 2/3, anti-GAPDH and anti-α-SMA antibody (Santa Cruz Bio-technology, Inc., Santa Cruz, CA) were used as the primary antibodies. Secondary antibodies were conjugated with horseradish peroxidase (Bio-Rad Laboratories). The signals were visualized by an enhanced chemiluminescence system (ECL, Amersham).

### Hepatic hydroxyproline content

Hydroxyproline content was determined as reported with slight modification [25,26]. Briefly, 100 mg of liver sample were hydrolyzed in 6 M HCl at 110°C for 24 h. After centrifugation at 2000 rpm at 48°C for 5 min, 2 ml of supernatant was mixed with 50 ml of 1% phenolphthalein and 8 N KOH to pH7–8. A 5 ml sample was subjected to a spectrophotometer at 560 nm to determine the content of hydroxyproline.

### Biochemical analysis of plasma

Samples of 1 ml blood were gathered from the retro-orbital plexus of each mouse and immediately centrifuged at 1,300 *g *at 4°C while plasma was kept at -20°C for liver function tests. ALT levels were measured using a colorimetric analyzer [27] (Dri-Chem 3000, Fuji Photo Film Co, Tokyo, Japan).

### Histological and immunohistochemical analysis of TGF-β1 and α-SMA expression

Mouse liver tissues were embedded in an optimal cutting temperature (OCT) compound (Miles Inc., Elkhart, IN) and frozen in liquid nitrogen. Five μm-thick cryosections were made by using cryostats (Leica CM 1800, Nussloch, Germany). The sections were fixed with cold acetone and endogenous peroxidase was inhibited by 3% H_2_O_2 _in phosphate buffered saline (PBS). Then the sections were incubated with 5% blocking serum (normal serum of the species of the secondary antibody). For modeling the negative control sections, the primary antibodies were substituted for the appropriate classes and isotypes of normal immunoglobulins (Igs). Controls for nonspecific binding of the secondary antibody were performed by replacing the solutions of the first step with PBS buffer. TGF-β1 was revealed by using a rabbit polyclonal antibody (Santa Cruz Biotechnology, Inc., Santa Cruz, CA), and an anti-rabbit IgG conjugated with Alexa Fluor 594 (Molecular Probes, Eugene, OR). For the detection of α-SMA, a mouse monoclonal antibody was used. Signals were visualized by anti-mouse IgG-HRP, horseradish peroxidase labeled secondary antibody, and 3, 3'-diaminobenzidine substrate (Vector Laboratories, Burlingame, CA). All sections were viewed under a microscope (Leica Mikrosysteme Vertrieb GmbH, Bensheim, Germany) [21,25].

### Masson's trichrome staining

Liver specimens were preserved in 4% paraformaldehyde in phosphate-buffered saline and dehydrated in a graded alcohol series. Following xylene treatment, the specimens were embedded in paraffin blocks and cut into 5 μm-thick sections stained with Masson's trichrome as described [21,25].

### Quantitative real-time reverse transcription-polymerase chain reaction (RT-PCR) analysis of collagen expression

Total RNA was isolated from the liver tissue by TRIZOL reagent (Invitrogen, Carlsbad, CA, USA). RT was performed as described [28]. Quantitative real-time PCR was performed with ABI Prism 7700 Sequence Detection System (Applied Biosystems, Foster City, CA). One μg of cDNA was used in each PCR reaction. The housekeeping GAPDH was used as a reference gene for normalization, and H_2_O was used as a negative control. The primers for the PCR reactions of Col 1A2 U08020 were: 5'-ACCTGTGTGTTCCCTACTCA-3' and 5'-GACTGTTGCCTTCGCCTC TG-3', the reaction was catalyzed by Taq polymerase (Invitrogen Corp, Carlsbad, CA). SYBR Green I DNA-binding dye generated the fluorescence signals during each of the 35 cycles, in proportion to the quantities of double-stranded DNA (denaturation 15 s at 95°C, annealing 15 s at 56°C and extension 40 s at 72°C). Each sample was analyzed in triplicate. Detection of the PCR products by agarose gel electrophoresis confirmed the homogeneity of the DNA products. Relative quantitation was calculated using the comparative threshold cycle (C_T_) method [as described in the User Bulletin #2, ABI PRISM 7700 Sequence Detection System]. Relative quantification of the Col 1A2 transcript was compared to that of the untreated negative control by the following formula: 2−Δ(ΔCT)
 MathType@MTEF@5@5@+=feaafiart1ev1aaatCvAUfKttLearuWrP9MDH5MBPbIqV92AaeXatLxBI9gBaebbnrfifHhDYfgasaacH8akY=wiFfYdH8Gipec8Eeeu0xXdbba9frFj0=OqFfea0dXdd9vqai=hGuQ8kuc9pgc9s8qqaq=dirpe0xb9q8qiLsFr0=vr0=vr0dc8meaabaqaciaacaGaaeqabaqabeGadaaakeaacqaIYaGmdaahaaWcbeqaaiabgkHiTiabfs5aejabcIcaOiabfs5aejabboeadnaaBaaameaacqqGubavaeqaaSGaeiykaKcaaaaa@35AA@ and calculated ΔC_T Col 1A2 _= C_T Col 1A2 _- C_T GAPDH _and Δ(ΔC_T_) = ΔC_T Col 1A2 _- ΔC_T negative control _[29].

### Preparation of nuclear extracts and EMSA

The preparation of liver nuclear extracts was based on the method described by Chang and Huang with minor modifications [30]. Fresh liver tissue of 0.1 g was homogenized with a Polytron (Kinematica) in 1 ml of buffer A (10 mM HEPES (pH 7.9), 1.5 mM magnesium chloride, 10 mM potassium chloride, 0.5 mM phenylmethylsulfonyl fluoride, 0.5 mM dithiothreitol, 2 μg/ml leupeptin, 10 μg/ml aprotinin, 50 mM sodium fluoride, and 1 mM sodium orthovanadate), incubated on ice for 10 min and then gently shaken for 10 s. The pellet of the crude nuclei was collected by centrifugation at 12,000 *g *for 10 s, resuspended in 300 μl of buffer C (20 mM HEPES (pH 7.9), 25% glycerol, 420 mM sodium chloride, 1.5 mM magnesium chloride, 0.2 mM EDTA, 0.5 mM phenylmethylsulfonyl fluoride, 0.5 mM dithiothreitol, 2 μg/ml leupeptin, 10 μg/ml aprotinin, 50 mM sodium fluoride, and 1 mM sodium orthovanadate) by vortex for 15 s, and then incubated on ice for 20 min. After centrifugation at 12,000 *g *for 2 min, the supernatant containing the nuclear proteins was collected, quantified with BCA Protein Assay Reagent (Pierce), and stored at -70°C in aliquots. For EMSA assay we used the following oligonucleotides: consensus Sp1 (f) 5'-GTT GCG GGG CGG GGC CGA GTG-3' and consensus Sp1 (r) 3'-AAC GCC CCG CCC CGG CTC ACG-5' and labeled the probes with ^32^P-dCTP by fill-in method [30].

### Statistics

Results were displayed by means of mean ± SD. Statistical analysis was carried out by F-test (for confirming homogeneity of variances) and two-tailed Student's *t*-test (for evaluating differences between means). *P *values lower than 0.05 (*) and 0.01 (**) were considered statistically significant.

## Competing interests

The author(s) declare that they have no competing interests.

## Authors' contributions

KLY performed most of the experiments and drafted the manuscript. KCH participated in the design of the study. WTC performed the EMSA and edited the manuscript. EL coordinated the study and finally edited the manuscript. All authors have read and approved the content of the manuscript.

## Supplementary Material

Additional file 1 Liver sections of CCl_4_-induced fibrosis. **Comparative histology of liver from mice treated with carbon tetrachloride (CCl_4_) and hydrodynamics-based transfer TGF-β gene**. Liver sections were stained with Masson's trichrome. (A) Mice were injected intraperitoneally with 0.3 ml CCl_4 _solution (4% CCl_4 _in corn oil) twice per week for 8 weeks. (B) Ten μg of plasmid (pPK9a) was dissolved in 3.0 ml Ringer's solution and injected into the mouse tail vein in a short duration of 5–7 s. The mice were fed water containing 25 mM ZnSO_4_*ad libitum*_. _The collagen fibers peaked at day 2 indicated by arrows. Bar = 500 μm. Click here for file
