# Supplementary figures and images for: Establishment of an early liver fibrosis model by the hydrodynamics-based transfer of TGF-β1 gene
Source: Comp Hepatol. 2007 Oct 19;6:9. doi: 10.1186/1476-5926-6-9 (PMC2104536; doi:10.1186/1476-5926-6-9)

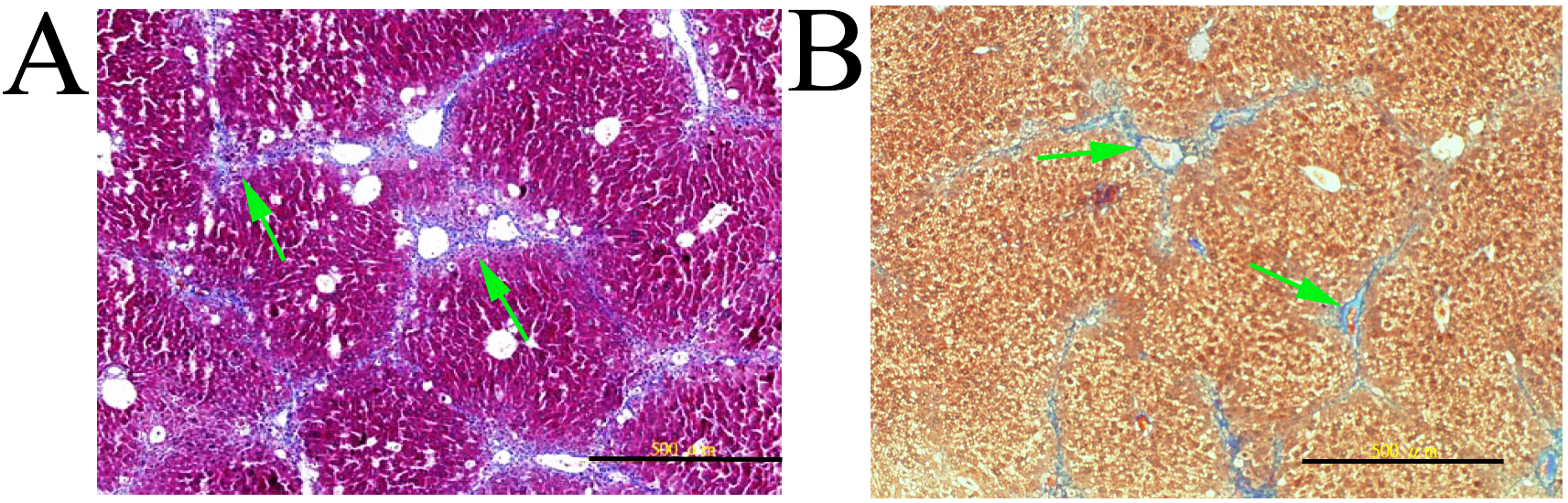

Supplement: Additional file 1 — Liver sections of CCl4-induced fibrosis. Comparative histology of liver from mice treated with carbon tetrachloride (CCl4) and hydrodynamics-based transfer TGF-β gene. Liver sections were stained with Masson's trichrome. (A) Mice were injected intraperitoneally with 0.3 ml CCl4 solution (4% CCl4 in corn oil) twice per week for 8 weeks. (B) Ten μg of plasmid (pPK9a) was dissolved in 3.0 ml Ringer's solution and injected into the mouse tail vein in a short duration of 5–7 s. The mice were fed water containing 25 mM ZnSO4ad libitum. The collagen fibers peaked at day 2 indicated by arrows. Bar = 500 μm. [file 1476-5926-6-9-S1.tiff]
